# The Dietary Reference Intakes for Japanese (2025): Overview and Future Directions for the Pregnant and Lactating Women's Section

**DOI:** 10.1111/jog.70236

**Published:** 2026-03-12

**Authors:** Yoshifumi Kasuga, Takashi Sugiyama, Satoshi Sasaki, Keiko Asakura

**Affiliations:** ^1^ Department of Obstetrics and Gynecology Keio University School of Medicine Tokyo Japan; ^2^ Department of Obstetrics and Gynecology Ehime University Graduate School of Medicine Ehime Japan; ^3^ Department of Social and Preventive Medicine, School of Public Health, Graduate School of Medicine The University of Tokyo Tokyo Japan; ^4^ Department of Preventive Medicine, School of Medicine Toho University Tokyo Japan

**Keywords:** diet, energy, lactation, nutrients, pregnancy

## Abstract

**Aim:**

Guided by the Developmental Origins of Health and Disease framework, maternal nutrition influences perinatal outcomes and lifelong offspring health. This commentary summarizes the pregnant and lactating women's section of Dietary Reference Intakes for Japanese (2025), contextualizes them within current Japanese evidence, and identifies priorities for future research and revisions.

**Methods:**

We conducted a narrative synthesis of the Dietary Reference Intakes and recent Japanese studies on maternal diet, fetal growth, anemia, folate, vitamin D, calcium, gestational weight gain, lactation, and preconception health, with attention to guideline‐practice gaps.

**Results:**

The Dietary Reference Intakes provide trimester‐based additional energy and nutrient targets. However, Japanese cohort data show suboptimal intakes of energy, macro‐ and micronutrients, with persistently low folate awareness and intake. Anemia (Hb < 11 g/dL) is associated with adverse outcomes, and low vitamin D/calcium status impacts maternal bone health. While medical nutrition therapy is standard for gestational diabetes, nutrient targets have not been set yet. Guidance for hypertensive disorder of pregnancy recommends sodium restriction but lacks comprehensive nutrient specifications. Evidence gaps include body mass index‐specific energy needs for appropriate gestational weight gain, robust diet–fetal growth relationships, and actionable guidance for lactation and the preconception period.

**Conclusions:**

The Dietary Reference Intakes for Japanese (2025) provide a necessary framework, yet significant implementation and evidence gaps remain. Research defining body mass index‐stratified energy needs, nutrient–outcome dose responses, and preconception/lactation guidance, paired with education and surveillance, is critical for improving intergenerational health.

## 1 Introduction

Considering the developmental origins of health and disease, the intrapartum environment is important for perinatal outcomes and the long‐term health of the offspring [1]. Factors affecting the intrapartum environment include smoking, alcohol, medicine, drugs, stress, and nutrition. In Japan, the incidence of preterm birth (gestational weeks at birth < 37 weeks) is low, whereas the incidence of low birth weight (LBW; birth weight < 2500 g) is higher than that in other developed countries [2]. One of the reasons for LBW at term or late preterm in Japan is pre‐pregnancy underweight (pre‐pregnancy body mass index [BMI] < 18.5 kg/m^2^) and inadequate gestational weight gain (GWG) [2, 3]. Therefore, pre‐pregnancy malnutrition and strict management of GWG could influence fetal growth [4]. Given this background, the GWG recommendations to reach an appropriate gestational age were published by the Japan Society of Obstetrics and Gynecology (JSOG) in 2020 (Table 1). Subsequently, Takeda et al. reported that the recommended GWG was associated with the lowest risk of disease onset, confirming the validity of the current recommendation [5]. Although data suggest a reduction in the incidence of LBW [6], it is important to optimize maternal nutrition to improve the health of the next generation.

**TABLE 1:** Gestational weight gain recommendations by JSOG Perinatal Committee 2020.

Pre‐pregnancy BMI category	BMI	Gestational weight gain (kg)
Underweight	< 18.5	12–15
Normal weight	18.5–25	10–13
Overweight	25–30	7–10
Obese	≥ 30	Individually managed (up to 5 kg is a rough guide)

Abbreviations: BMI: body mass index; JSOG: Japan Society of Obstetrics and Gynecology.

Establishing optimal nutrient recommendations for pregnant and lactating women is difficult. Food Frequency Questionnaires (FFQs), which are often used in dietary assessments, may underestimate nutrient intakes; therefore, discrepancies between estimated and actual dietary intakes are assumed. Furthermore, conducting intervention studies among pregnant women to determine appropriate recommendations is challenging for ethical reasons. According to a large cohort study by the Japan Environment and Children's Study that used an FFQ, intakes of energy, macronutrients, and micronutrients before and during pregnancy are lower than those recommended for Japanese women [7]. However, because GWG was not reported, whether nutrient intakes are insufficient to support maternal health and appropriate fetal growth remains unclear.

From the perspective of individual nutrients, it has been reported that Japanese women do not consume sufficient folate to prevent neural tube defects (NTD) [8]. Some reports have indicated that many women do not recognize the importance of folic acid intake [9, 10, 11]. Anemia is one of the most important issues in perinatal care, and iron is considered the most important nutrient for anemia. Hemoglobin level < 11 g/dL is associated with LBW, very LBW, preterm birth, small for gestational age (SGA: birth weight < 10th percentile), stillbirth, perinatal mortality, and neonatal mortality [12]. In the United Kingdom, anemia in the first trimester has been associated with fetal congenital heart disease [13]. Calcium and vitamin D levels are also important during pregnancy and lactation. Particularly, adequate intake of these nutrients is critical to prevent pregnancy‐ and lactation‐associated osteoporosis and to support maternal and neonatal health [14]. Other nutrients likewise play an important role in improving maternal and neonatal outcomes. However, the importance of nutrition remains underappreciated among pregnant and lactating women and clinicians.

In 2005, the Dietary Reference Intakes for Japanese (DRI) were first published by the Ministry of Health, Labour and Welfare and have been updated every five years since then. The DRI proposes reference values for energy and nutrient intake in the Japanese population to promote and maintain health; these reference values guide meal planning in hospitals, schools, and other settings. This report summarizes the pregnant and lactating women's section of the revised DRI (2025) (Tables 2, 3) and underscores the issues that should be considered in future revisions.

**TABLE 2:** Dietary reference intakes for Japanese pregnant women (2025).

Energy			Estimated energy requirement
Energy	(kcal/day)	1st trimester	+50
		2nd trimester	+250
		3rd trimester	+450

Nutrients				Estimated average requirement	Recommended dietary allowance	Adequate intake	Dietary goal
Protein	(g/day)		1st trimester	+0	+0	—	—
			2nd trimester	+5	+5	—	—
			3rd trimester	+20	+25	—	—
	(%energy)		1st trimester	—	—	—	13–20
			2nd trimester	—	—	—	13–20
			3rd trimester	—	—	—	15–20
Dietary fats	Dietary fats		(%energy)	—	—	—	20–30
	Saturated fatty acid		(%energy)	—	—	—	≤ 7
	n‐6 fatty acid		(g/day)	—	—	9	—
	n‐3 fatty acid		(g/day)	—	—	1.7	—
Carbohydrates	Carbohydrates		(%energy)	—	—	—	50–65
	Dietary fiber		(g/day)	—	—	—	≥ 18
Vitamin	Fat‐soluble	Vitamin A (μgRAE/day)	1st & 2nd trimester	+0	+0	—	—
			3rd trimester	+60	+80	—	—
		Vitamin D	(μg/day)	—	—	9.0	—
		Vitamin E	(mg/day)	—	—	5.5	—
		Vitamin K	(μg/day)	—	—	150	—
	Water‐soluble	Vitamin B1	(mg/day)	+0.1	+0.2	—	—
		Vitamin B2	(mg/day)	+0.2	+0.3	—	—
		Niacin	(mgNE/day)	+0	+0	—	—
		Vitamin B6	(mg/day)	+0.2	+0.2	—	—
		Vitamin B12	(μg/day)	—	—	4.0	—
		Folate (μg/day)	1st trimester	+0	+0	—	—
			2nd & 3rd trimester	+200	+240	—	—
	
		Pantothenic acid	(mg/day)	—	—	5	—
		Biotin	(μg/day)	—	—	50	—
Vitamin C	(mg/day)	+10	+10	—	—
Minerals	Macrominerals	Sodium	(mg/day)	600	—	—	—
		Salt equivalent	(g/day)	1.5	—	—	< 6.5
		Potassium	(mg/day)	—	—	2000	≥ 2600
		Calcium	(mg/day)	+0	+0	—	—
		Magnesium	(mg/day)	+30	+40	—	—
		Phosphorus	(mg/day)	—	—	800	—
	Microminerals	Iron (mg/day)	1st trimester	+2.0	+2.5	—	—
			2nd & 3rd trimesters	+7.0	+8.5	—	—
		Zinc (mg/day)	1st trimester	+0	+0	—	—
			2nd & 3rd trimesters	+2.0	+2.0	—	—
		Copper	(mg/day)	+0.1	+0.1	—	—
		Manganese	(mg/day)	—	—	3.0	—
		Iodine	(μg/day)	+75	+110	—	—
		Selenium	(μg/day)	+5	+5	—	—
		Chromium	(μg/day)	—	—	10	—
		Molybdenum	(μg/day)	+0	+0	—	—

**TABLE 3:** Dietary reference intakes for Japanese lactating women (2025).

Energy				Estimated energy requirement			
Energy	(kcal/day)			+350			

Nutrients				Estimated average requirement	Recommended dietary allowance	Adequate intake	Dietary goal
Protein	(g/day)		(g/day)	+15	+20	—	—
	(%energy)		(%energy)	—	—	—	15–20
Dietary fats	Dietary fats		(%energy)	—	—	—	20–30
	Saturated fatty acid		(%energy)	—	—	—	≤ 7
	n‐6 fatty acid		(g/day)	—	—	9	—
	n‐3 fatty acid		(g/day)	—	—	1.7	—
Carbohydrates	Carbohydrates		(%energy)	—	—	—	50–65
	Dietary fiber		(g/day)	—	—	—	≥ 18
Vitamin	Fat‐soluble	Vitamin A	(μgRAE/day)	+300	+400	—	—
		Vitamin D	(μg/day)	—	—	9.0	—
		Vitamin E	(mg/day)	—	—	5.5	—
		Vitamin K	(μg/day)	—	—	150	—
	Water‐soluble	Vitamin B1	(mg/day)	+0.2	+0.2	—	—
		Vitamin B2	(mg/day)	+0.5	+0.6	—	—
		Niacin	(mgNE/day)	+3	+3	—	—
		Vitamin B6	(mg/day)	+0.3	+0.3	—	—
		Vitamin B12	(μg/day)	—	—	4.0	—
		Folate	(μg/day)	+80	+100	—	—
		Pantothenic acid	(mg/day)	—	—	6	—
		Biotin	(μg/day)	—	—	50	—
		Vitamin C	(mg/day)	+40	+45	—	—
Minerals	Macrominerals	Sodium	(mg/day)	600	—	—	—
		Salt equivalent	(g/day)	1.5	—	—	< 6.5
		Potassium	(mg/day)	—	—	2000	≥ 2600
		Calcium	(mg/day)	+0	+0	—	—
		Magnesium	(mg/day)	+0	+0	—	—
		Phosphorus	(mg/day)	—	—	800	—
	Microminerals	Iron	(mg/day)	+1.5	+2.0	—	—
		Zinc	(mg/day)	+2.5	+3.0	—	—
		Copper	(mg/day)	+0.5	+0.6	—	—
		Manganese	(mg/day)	—	—	3.0	—
		Iodine	(μg/day)	+100	+140	—	—
		Selenium	(μg/day)	+15	+20	—	—
		Chromium	(μg/day)	—	—	10	—
		Molybdenum	(μg/day)	+2.5	+3.5	—	—

## 2 Definitions

For adults (excluding pregnant and lactating women), the following equation is used to calculate the estimated energy requirement (EER):

$$\begin{array}{c} \mathrm{EER}=\text{basal metabolism reference value}\;\left(\text{kcal}/\mathrm{kg},\text{body weight}/\mathrm{day}\right)\\ \;\times \text{reference body weight}\;\left(\mathrm{kg}\right)\times \text{physical activity level} \end{array}$$

The EERs of infants, children, pregnant women, and lactating women are calculated by adding the amount of energy that is necessary for growth, the continuation of pregnancy, or lactation.

The estimated average requirement (EAR) is set to prevent inadequate nutrient intake and represents the intake level that meets the nutritional needs of 50% of the population. The recommended dietary allowance (RDA) supplements the EAR, and the intake level meets the nutritional needs of most (97%–98%) populations. When scientific evidence is insufficient to establish an EAR and RDA, an adequate intake (AI) is defined instead. Since AI is the intake amount required to maintain adequate nutrients, ensuring that dietary intakes do not fall below the AI minimizes the risk of nutritional deficiency. The upper intake level (UL) is the highest average daily nutrient intake level that is not likely to pose any risk of adverse health effects. As the intake exceeds the UL, the potential risk of adverse health effects may increase. If the evidence is insufficient, a UL is not set. Notably, any ULs are not currently set for pregnant or lactating women. Tentative Dietary goals for Preventing Lifestyle‐related Diseases (DGs) indicate the nutrient intake amount that Japanese people should aim for in the foreseeable future to reduce lifestyle‐related diseases.

## 3 Pregnant Women

For pregnant women, the additional EER was calculated as follows: change in total energy expenditure due to pregnancy (kcal/day) + energy disposition (kcal/day). This value reflects the energy requirement of non‐pregnant women of childbearing age. Thus, the additional EER was calculated on the basis of the energy required for non‐pregnant women to gain 11 kg of gestational weight by 40 weeks. Given that the current EER is not supported by data derived from pregnant women, it might be considered inadequate from a physiological and metabolic perspective.

Furthermore, in 2020, the GWG recommendations were published by the JSOG (Table 1). Although GWG is determined by the pre‐pregnancy BMI category, EER is not classified by BMI category in the DRI 2025. Since the Japanese GWG percentile charts for every pre‐pregnancy BMI category have been published [15], it was considered that the EER might be set to reach the appropriate GWG. However, further research is required because there are no reports examining the energy intake required to achieve an appropriate GWG for each pre‐pregnancy BMI category. Additionally, since nutritional education for underweight pregnant women ensures appropriate GWG and improves fetal outcomes [16], the importance of nutrition should be emphasized for women of every childbearing age.

The nutrient recommendations for pregnant women were set by considering the requirements for non‐pregnant women of the same age. Changes during pregnancy were also considered. In a retrospective cohort study that analyzed the association between birth weight and maternal dietary intake during pregnancy, Eshak et al. reported that energy, dietary fiber, carbohydrates, and vitamins were associated with fetal growth [17]. Morisaki et al. reported that the highest birth weight and the lowest incidence of SGA was attained when the percentage energy from protein was 12% whereby the association between the percentage energy from protein and fetal growth was an inverse U‐shape [18]. However, few studies have investigated the association between fetal growth and nutrient intake, especially in the Japanese population. This is because it is ethically difficult to perform a randomized controlled study and an intervention study in pregnant women to determine the effects of nutrient intake.

Furthermore, some pregnant women may develop several perinatal complications (e.g., emesis, gestational diabetes [GDM], and hypertensive disorder of pregnancy [HDP]). In Japan, GDM is managed with diet and/or insulin therapy [19]. In the nutritional recommendations for GDM in several countries, including Japan, although glucose targets; the ratio of carbohydrates, lipids, and proteins; and the intake of folic acid and vitamin D were described, other nutrients were not set [20]. In mothers with HDP, although the Japanese HDP guidelines recommend salt‐intake reduction, neither Japan nor other countries have established recommendations for the intake of other nutrients.

Primarily, the DRI comprises recommendations for people without any complications. However, to prevent perinatal complications, it is relevant to specify energy and nutrient intake recommendations as this could improve both short‐ and long‐term healthcare for mothers and offspring.

## 4 Lactating Women

For lactating women, the additional EER was calculated from the energy consumed to synthesize breast milk. The recommended additional EER is +350 kcal/day. The Children and Families Agency in Japan reports that for infants under 2 months, feeding methods were distributed as follows: exclusive breastfeeding (34.5%), formula milk (11.7%), and combination feeding (53.8%). Additionally, 53.1% of infants completed weaning by their first birthday.

Vitamin D and 25‐hydroxyvitamin D levels in breastfeeding infants in the summer of 2016–2017 were lower than those in 1989 [21]. Vitamin D and calcium are important nutrients that help prevent osteoporosis associated with pregnancy and lactation. Yoshitaka et al. reported that lower 25‐hydroxyvitamin D (< 8.9 ng/mL in the first trimester) levels were associated with reduced bone mineral density (BMD) in mothers with a first‐trimester BMI < 20 kg/m^2^, but not in those with a BMI ≥ 20 kg/m^2^ [22]. Furthermore, Sho et al. suggested that calcium intake from milk products may be prevented by decreasing BMD [23]. In a study on maternal milk in 71 lactating Japanese mothers 1 month after delivery, retinol intake was associated with carbohydrate content, and energy was associated with neonatal growth. However, the macronutrients in maternal milk were not associated with neonatal growth [24]. As lactation behavior is determined by maternal, neonatal, socioeconomic, and lifestyle factors [25], it may prove more difficult to specify recommendations for lactating women than for pregnant women. Since Nojiri et al. commenced a prospective study to reveal the association between lactation and offspring's growth, further studies undertaken using this dataset may facilitate the development of nutritional recommendations for lactating mothers [26]. Despite several reports on nutrients that are essential for the health of lactating women and infant growth, there is a lack of strong evidence to support recommendations for nutrient intake, and further evidence is required.

## 5 Preconception Care for Nutrition

Preconception care is a key factor for achieving good perinatal prognosis, and maternal nutrition before conception affects perinatal outcomes and fetal growth. However, recommendations for the pre‐conception care period were not included in the DRI 2025. Particularly, folate intake before pregnancy is more important than folate intake during pregnancy. The C677T mutation in methylenetetrahydrofolate reductase (*MTHFR*) is associated with folate metabolism. The frequency of the TT genotypes of *MTHFR* in the Japanese population is approximately 15%, which is higher than that in other ethnicities [27]. Although the C677T mutation in Asian non‐Japanese and Caucasian individuals confers a risk for NTDs [28, 29, 30], data are scarce on the association between *MTHFR* genotype and NTDs in the Japanese population. Despite the awareness regarding the importance of folate intake, the incidence of NTDs has not decreased. Although the C677T mutation might be associated with the development of NTDs in the Japanese population, a small‐sample analysis by Kondo et al. indicated the irrelevance of testing for the C677T variant in *MTHFR* because it was not associated with NTDs in Japanese individuals [31]. Thus, further research using Japanese large‐sample genetic data is required to investigate the association between the *MTHFR* genotype and NTDs. Fukuda et al. reported that dietary folate intake was positively associated with the plasma folate level in young Japanese women with the CC or CT, but not TT, genotype in *MTHFR* [32]. As urinary potassium excretion might be a good predictor of folate intake, according to Sakurada et al., the monitoring of folate intake using urinary potassium excretion could be useful in predicting adverse outcomes owing to folate deficiency [33]. Therefore, pre‐conceptional testing for the C677T variation and urinary potassium excretion might enable more effective guidance on their dietary folate intake.

Additionally, a high‐quality diet before pregnancy might prevent HDP according to a study that evaluated dietary patterns using the Balanced Diet Score and the Dietary Approaches to Stop Hypertension score in Japanese women [34]. In another report evaluating the association between a healthy lifestyle before pregnancy and adverse pregnancy outcomes (APOs), including GDM, HDP, preterm birth, LBW, and SGA, pregnant women with higher health‐life scores had a lower risk of APOs [35]. Preventing adverse perinatal outcomes could therefore benefit maternal and offspring health. Future DRI should formally incorporate preconception care for Japanese people.

It has been suggested that preconception education interventions may be useful in improving maternal health [36]. However, data on men's preconception educational intervention are scarce [37]. Recent studies have highlighted the effect of paternal nutritional status on APOs and the offspring's future health [38]. Therefore, to enhance awareness regarding the importance of nutritional preconception care for men, the DRI should include recommendations for the preconception care of not only women but also men.

## 6 Conclusion

The DRI 2025 provides the necessary framework; however, gaps in implementation and evidence remain. Research defining BMI‐stratified energy needs, nutrient–outcome dose response, and preconception/lactation guidance, paired with education and surveillance, will be critical for improving intergenerational health.

## Author Contributions

**Yoshifumi Kasuga:** writing – original draft, writing – review and editing, project administration. **Takashi Sugiyama:** writing – review and editing. **Satoshi Sasaki:** writing – review and editing. **Keiko Asakura:** writing – review and editing, project administration, funding acquisition.

## Funding

This work was supported by Health Labour Sciences Research Grant (24FA1022) awarded to Keiko Asakura.

## Disclosure

The authors declare no conflicts of interest. Yoshifumi Kasuga is an Editorial Board member of *The Journal of Obstetrics and Gynecology Research*. To minimize bias, he was excluded from all editorial decision making related to the acceptance of this article for publication.

## Consent

The authors have nothing to report.

## Conflicts of Interest

The authors declare no conflicts of interest.

## Data Availability

Data sharing not applicable to this article as no datasets were generated or analysed during the current study.
